# Role of Autophagy in Male Reproductive Processes in Land Plants

**DOI:** 10.3389/fpls.2020.00756

**Published:** 2020-06-17

**Authors:** Takuya Norizuki, Naoki Minamino, Takashi Ueda

**Affiliations:** ^1^Department of Biological Sciences, Graduate School of Science, The University of Tokyo, Tokyo, Japan; ^2^Division of Cellular Dynamics, National Institute for Basic Biology, Okazaki, Japan; ^3^The Department of Basic Biology, SOKENDAI (The Graduate University for Advanced Studies), Okazaki, Japan

**Keywords:** autophagy, male reproductive processes, tapetum, pollen germination, spermiogenesis

## Abstract

Autophagy is a highly conserved system for degrading and recycling cytoplasmic components. The identification of autophagy-related (*ATG*) genes, required for autophagosome formation, has led to numerous studies using *atg* mutants. These studies have revealed the physiological significance of autophagy in various functions of diverse organisms. In land plants, autophagy is required for higher-order functions such as stress responses and development. Although defective autophagy does not result in any marked defect in the reproductive processes of *Arabidopsis thaliana* under laboratory conditions, several studies have shown that autophagy plays a pivotal role in male reproduction in several land plants. In this review, we aim to summarize information on the role of autophagy in male reproductive processes in land plants.

## Introduction

**Figure 1:**
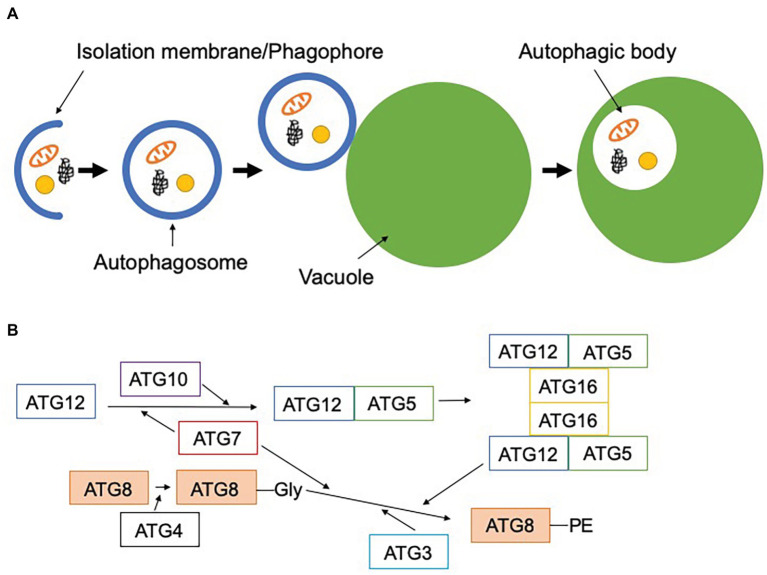
Scheme of macroautophagy. **(A)** Macroautophagy starts with the formation of the isolation membrane (phagophore) in the cytosol. This engulfs cytoplasmic components and forms the double membrane-bound autophagosome. The outer membrane of the autophagosome fuses with the vacuolar membrane to release a single membrane-bound autophagic body into the vacuole. **(B)** Two ubiquitin-like conjugation systems are involved in the lipidation of ATG8. First, ATG12 is conjugated to ATG5 by ATG7 (E1-like) and ATG10 (E2-like), and ATG12-ATG5 forms a complex with ATG16. ATG8 is cleaved by ATG4, resulting in the exposure of glycine at its carboxyl terminus. This processed ATG8 is conjugated to phosphatidylethanolamine by ATG7 (E1-like), ATG3 (E2-like), and the dimeric ATG12-ATG5-ATG16 complex (E3-like). Lipidated ATG8 can be localized to the autophagosomal membrane.

Autophagy is a highly conserved system for degrading and recycling cytoplasmic components, including organelles, in the vacuole or lysosome. Among the various modes of autophagy reported thus far, macroautophagy, hereafter referred to as autophagy, has been the most intensively studied. This type of autophagy begins with the formation of a membrane sac called the isolation membrane (also known as the phagophore), which extends, engulfing cytoplasmic components, to form a double membrane-bound autophagosome. The outer membrane of this autophagosome fuses with the vacuolar membrane, releasing the inner membrane-bound autophagic body into the vacuolar lumen, to be degraded by vacuolar hydrolases (Figure 1A; Takeshige et al., 1992; Baba et al., 1994). In the 1990s, a gene set required for autophagosome formation, hereafter referred to as core autophagy-related (*ATG*) genes, was identified by forward genetics in *Saccharomyces cerevisiae* (Tsukada and Ohsumi, 1993; Thumm et al., 1994; Harding et al., 1995; Klionsky et al., 2003). The core *ATG* genes encode a group of Atg proteins that form several functional units: the Atg1 complex, the phosphatidylinositol 3-kinase (PI3K) complex, Atg9, the Atg2-Atg18 complex, and two ubiquitin-like conjugation system complexes (Nakatogawa et al., 2009; Mizushima et al., 2011). One of the core Atg proteins, Atg8, is conjugated to phosphatidylethanolamine by the ubiquitin-like conjugation systems (Figure 1B; Mizushima et al., 1998, 1999; Kirisako et al., 1999, 2000; Shintani et al., 1999; Ichimura et al., 2000; Hanada et al., 2007). Since lipidated Atg8 localizes to the isolation membrane from the beginning until after completion of autophagosome formation, it is commonly used as an autophagosome marker in various organisms (Kirisako et al., 1999; Kabeya et al., 2000; Yoshimoto et al., 2004). Reverse genetic approaches have unraveled the physiological roles of autophagy in a wide range of biological functions, including metabolic adaptation, intracellular quality control, and development (Mizushima and Komatsu, 2011; Mizushima, 2018).

In land plants (embryophytes), core *ATG* genes are highly conserved, and their functions are shown to be similar to homologs in yeast and mammals (Avin-Wittenberg et al., 2012; Yoshimoto, 2012; Norizuki et al., 2019). Studies of *Arabidopsis thaliana atg* mutants have demonstrated that autophagy is involved in responses to abiotic and biotic stressors such as nutrient starvation and pathogen attacks (Marshall and Vierstra, 2018). Furthermore, recent studies have shown that autophagy plays a critical role in male reproduction in various species including *Oryza sativa*, *Nicotiana tabacum*, *Marchantia polymorpha*, and *Physcomitrella patens* (Kurusu et al., 2014; Minamino et al., 2017; Sanchez-Vera et al., 2017; Zhao et al., 2020). In this review, we will briefly outline male reproduction in angiosperms and bryophytes, then summarize the physiological roles of autophagy in these processes.

## Male Reproduction in Land Plants

**Figure 2:**
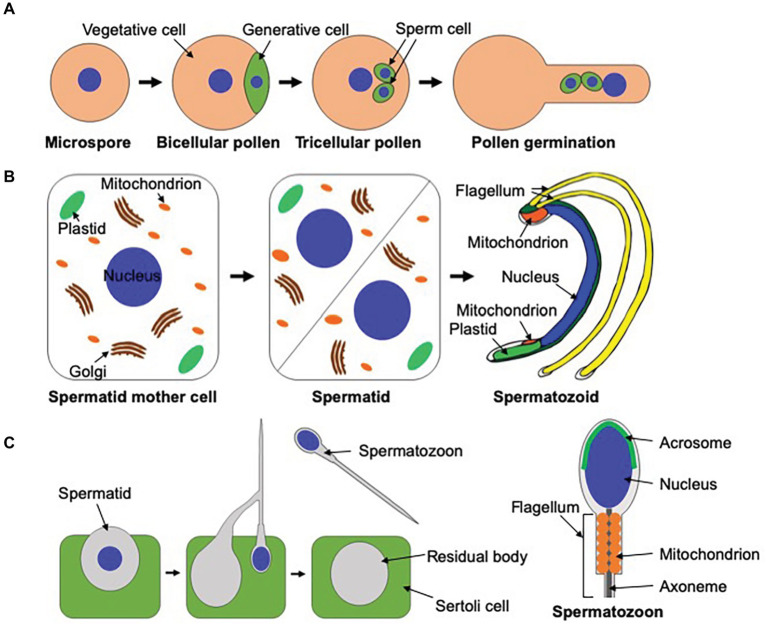
Male gametogenesis in *Arabidopsis thaliana*, *Marchantia polymorpha*, and mammals. **(A)** In *A. thaliana*, microspores generated from meiosis of pollen mother cells undergo asymmetrical cell division to form vegetative and generative cells. Each generative cell divides symmetrically to yield two immotile sperm cells. Once pollen grains are attached to the surface of stigmas, they germinate to produce pollen tubes, which transport male gametes to female gametes. This figure is illustrated based on figures in Berger and Twell (2011) and Hackenberg and Twell (2019). **(B)** In *M. polymorpha*, spermatids are formed by the diagonal cell division of spermatid mother cells. Spermatids undergo a dynamic morphogenetic transformation called spermiogenesis to form spermatozoids. This figure was illustrated based on a figure in Shimamura (2016). **(C)** Dynamic cellular reorganization also takes place during mammalian spermiogenesis. Just before the release of spermatozoa, unnecessary cytoplasmic components are excluded from their cell bodies as the residual body, which is phagocytosed and degraded by the neiboring Sertoli cell.

Various organisms reproduce through a sexual process in which haploid male and female gametes fuse with each other to generate diploid zygotes. Male gametes in land plants are roughly classified into two types based on the presence or absence of flagella. In angiosperms and the majority of gymnosperms, male gametes lack a flagellum and are therefore immotile, requiring transportation to egg cells *via* pollen tubes to accomplish fertilization. Conversely, bryophytes, lycophytes, monilophytes, and some gymnosperms such as ginkgoes and cycads utilize motile male gametes called spermatozoids, which are equipped with two or more flagella, for sexual reproduction (Southworth and Cresti, 1997; Renzaglia and Garbary, 2001). In both cases, drastic reorganization of cellular components occurs during male gamete development (Hackenberg and Twell, 2019). In the angiosperm, *A. thaliana*, four haploid microspores are produced by meiosis of a diploid pollen mother cell. Each microspore divides asymmetrically to form vegetative and generative cells, and each generative cell undergoes symmetrical division to form two sperm cells (Figure 2A; Southworth and Russell, 2001; Berger and Twell, 2011). Once pollen grains are attached to the surface of stigmas, they germinate to produce pollen tubes, which are precisely guided to female gametes to deliver sperm cells (Figure 2A; Higashiyama and Takeuchi, 2015; Zheng et al., 2018). Pollen grains are covered by an outer cell wall called the exine, which provides chemical and physical protection against stressors. The tapetum surrounding pollen grains plays a pivotal role in the synthesis of the exine by supplying nutrients and metabolites to pollen grains (Ariizumi and Toriyama, 2011).

In contrast to angiosperms, in which the sporophytic generation is dominant in the life cycle, the gametophytic generation is dominant in bryophytes, and spermatozoids are generated without meiosis. In the liverwort, *M. polymorpha*, spermatids are produced by the diagonal division of spermatid mother cells. Spermatids then differentiate into motile spermatozoids through a dynamic morphological conversion called spermiogenesis. This process includes *de novo* synthesis of the locomotory apparatus, chromatin condensation, nuclear elongation, a decrease in the number of mitochondria, and exclusion of a major part of the cytoplasm (Figure 2B). Spermatozoids move toward female gametes in water to accomplish fertilization (Shimamura, 2016). Although the molecular mechanisms of male reproduction in angiosperms are well-documented, molecular mechanisms of spermatozoid formation in basal land plants remain mostly ambiguous (Hackenberg and Twell, 2019).

## Role of Autophagy in Male Reproductive Development in Angiosperms

Studies of *A. thaliana atg* mutants have not detected a marked effect of *atg* mutations on sexual reproduction under normal experimental conditions, whereas these mutations affect vegetative growth in this species (Doelling et al., 2002; Hanaoka et al., 2002; Marshall and Vierstra, 2018). Mutation in *ATG6* is the only exception; the *atg6* mutant exhibits a defect in pollen germination (Fujiki et al., 2007; Qin et al., 2007; Harrison-Lowe and Olsen, 2008). However, this defect might not be a result of defective autophagy. In yeast, Atg6 is also known as Vps30 and forms a complex with Vps34 and Vps15 to produce phosphatidylinositol 3-phosphate (PI3P) from phosphatidylinositol (Kihara et al., 2001). Because the *A. thaliana vps15* mutant also exhibits a defect in pollen germination and PI3P is also required for various cellular reactions beyond autophagy, defective pollen germination in the *atg6* mutant could result from a deficiency independent of autophagy (Fujiki et al., 2007; Qin et al., 2007; Harrison-Lowe and Olsen, 2008; Xu et al., 2011; Wang et al., 2012). Thus, *ATG*-dependent autophagy should be dispensable for male reproduction in *A. thaliana*. In addition, *Zea mays atg* mutants are fertile under normal experimental conditions (Li et al., 2015). However, autophagy is indispensable for male reproduction in *O. sativa* (Kurusu et al., 2014). In this species, autophagy is highly activated in the tapetum during microspore development (Kurusu et al., 2014; Hanamata et al., 2019). The tapetum undergoes programmed cell death to supply metabolites and nutrients to developing microspores, which is essential for pollen maturation and pollen tube elongation (Ku et al., 2003; Kawanabe et al., 2006; Li et al., 2006; Zhang et al., 2008). The *atg7* mutant exhibits limited anther dehiscence, and its pollen maturation and germination are severely compromised, resulting in markedly reduced male fertility (Kurusu et al., 2014; Sera et al., 2019). This could be explained by the fact that the *atg7* mutant exhibits defective programmed cell death of the tapetum, which could result in an insufficient supply of metabolites and nutrients to developing microspores (Kurusu et al., 2014). Given that autophagy executes programmed cell death during tracheary element differentiation in *A. thaliana* and embryogenesis in *Picea abies* (Kwon et al., 2010; Minina et al., 2013), autophagy could directly induce programmed cell death in the tapetum of *O. sativa*. Alternatively, autophagy might indirectly affect programmed cell death by regulating the metabolism of phytohormones. The phytohormone gibberellin plays an essential role in the development of tapeta and pollen in *O. sativa* (Chhun et al., 2007; Aya et al., 2009). Gibberellin accumulation is reduced in the anther of the *atg7* mutant, and treatment with active gibberellin (GA_4_) fully and partially repairs the defect in pollen maturation and germination, respectively. This suggests that autophagy regulates the development of male reproductive tissues *via* the metabolism of gibberellin to some extent (Kurusu et al., 2017). The different effects of defective autophagy on male fertility between *O. sativa* and *A. thaliana* might result from differences in the structure of the tapetum, lipidic components of pollen grains, or both (Hanamata et al., 2014). Further study will be needed to clarify why autophagy is particularly required during male reproductive processes in *O. sativa*.

Cellular and molecular reorganization during pollen germination and pollen tube elongation also involve autophagy. In addition to the essential role of autophagy in *O. sativa* pollen germination described above (Kurusu et al., 2014), a similar process in *N. tabacum* also requires autophagy (Zhao et al., 2020). In this species, autophagy is highly activated during the initial stage of pollen germination, and autophagosomes accumulate around the germination aperture. *ATG2*, *ATG5*, and *ATG7* RNAi *N. tabacum* lines exhibit reduced rates of pollen germination, and in these lines, unlike in wild-type plants, a convex layer of the cytoplasm containing mitochondria remains at the germination aperture. Furthermore, a mitochondrial marker and the autophagosome marker ATG8 partially colocalize, and cardiolipin, a mitochondria-specific phospholipid, accumulates in the *ATG* RNAi lines. This information suggests that mitochondria are a target of autophagy in *N. tabacum* pollen grains (Zhao et al., 2020). In contrast, *atg* mutants of *A. thaliana* exhibit no detectable abnormality in pollen germination (Zhao et al., 2020). Vacuolar degradation systems other than *ATG*-dependent autophagy might contribute to reorganization of intracellular components during pollen germination in *A. thaliana*; this should be verified in future studies.

## Role of Autophagy During Bryophyte Spermiogenesis

The spermatozoids of most bryophytes consist of two flagella and a cell body, which comprises an elongated spiral nucleus, one plastid, two mitochondria, and trace amounts of cytosol (Figure 2B; Renzaglia and Garbary, 2001; Shimamura, 2016). Although reorganization of intracellular structures during bryophyte spermiogenesis has been intensively observed by transmission electron microscopy (TEM; Renzaglia and Garbary, 2001), the dynamics of intracellular reorganization remain unclear. The moss, *P. patens*, and the liverwort, *M. polymorpha*, are model plants associated with genetic studies (Rensing et al., 2008; Strotbek et al., 2013; Ishizaki et al., 2016; Bowman et al., 2017). Taking advantage of various organelle markers established in *M. polymorpha* (Kanazawa et al., 2016; Minamino et al., 2018), Minamino et al. (2017) observed the dynamics of organelles during spermiogenesis by confocal microscopy. They found that the size of the vacuole increases during spermiogenesis, and proteins in various organelles, including the plasma membrane, Golgi apparatus, and multivesicular endosomes, are transported to the luminal space of the vacuole during spermiogenesis. These findings indicate that the vacuole plays a major role in the removal and degradation of cellular components, including organelles, during *M. polymorpha* spermiogenesis (Minamino et al., 2017). Multivesicular endosomes and autophagosomes, which are involved in endocytic degradation of membrane proteins and degradation of cytoplasmic components, respectively, are frequently observed in spermatids undergoing spermiogenesis. The number of autophagosomes increases during spermiogenesis, and autophagic body-like structures are observed inside the vacuole, suggesting that autophagy is activated during spermiogenesis. These findings suggest that both autophagy and endocytic degradation play important roles during *M. polymorpha* spermiogenesis.

A critical role of autophagy in spermiogenesis has been identified in *P. patens* (Sanchez-Vera et al., 2017). Autolysosome-like structures are frequently observed in spermatids undergoing spermiogenesis; these may be formed by fusion between autophagosomes and the vacuole. An elevated expression level of GFP-PpATG8e has also been detected in *P. patens* spermiogenesis, suggesting upregulated autophagy during this process. Furthermore, spermatozoids of the autophagy-defective *atg5* mutant are sterile and possess a wide spectrum of morphological abnormalities such as a larger amount of cytoplasm and an abnormally shaped nucleus. TEM observation has also revealed that the *atg5* mutation impairs decreasing the number of mitochondria and plastids, and flagellar formation during spermiogenesis (Sanchez-Vera et al., 2017). Thus, autophagy plays an important role in male gametogenesis in bryophytes, whose molecular regulatory mechanisms would be interesting targets to study.

## Male Reproduction and Autophagy in the Mammalian System

Mammalian sexual reproduction also utilizes motile male gametes with a flagellum (spermatozoa; Figure 2C), whose composition of intracellular structures is different from that of bryophytes. A mammalian spermatozoon possesses a nucleus at its head and a flagellum at the tail, and a mitochondrial helical sheath surrounds the axoneme at the midpiece (Figure 2C; Toure et al., 2020). Although the exclusion of the cytoplasm takes place during spermiogenesis in both mammals and bryophytes, their molecular mechanisms must be not the same. Although the residual body released from mammalian spermatids, which contain unnecessary cytoplasmic components, is removed by the phagocytic activity of the neighboring Sertoli cell during mammalian spermiogenesis (O’Donnell et al., 2011), phagocytosis by neighboring cells cannot take place in bryophytes due to the surrounding rigid cell wall (Figure 2C). Nevertheless, autophagy also plays indispensable roles during mammalian spermiogenesis. The germ cell-specific *ATG7* knockout in mice results in male sterility, exhibiting multiple defects in spermiogenesis, such as defective biogenesis of the acrosome (Wang et al., 2014). The acrosome, which is not present in the male gametes of plants, is a lysosome-related organelle required for fertilization (Figure 2C; Moreno and Alvarado, 2006; Ikawa et al., 2010; Khawar et al., 2019). LC3, which is homologous to yeast Atg8, is localized on the proacrosomal vesicles in an *ATG7*-dependent manner. These proacrosomal vesicles accumulate near the nucleus without fusing with each other in the *atg7* mutant, suggesting that autophagy is required for the biogenesis of the acrosome (Wang et al., 2014). Another marked defect in the mouse *atg7* mutant is the abnormal reorganization of microtubules during spermiogenesis. Irregular cytoskeletal structures are observed in autophagy-defective mouse embryonic fibroblasts (MEFs). PDLIM1, a regulator of cytoskeletons, accumulates in *atg7* MEFs and spermatids, and knockdown of PDLIM1 partially suppresses cytoskeletal defects in *atg7* MEFs. These results suggest that autophagy regulates cytoskeletal organization by degrading PDLIM1 (Shang et al., 2016). The *P. patens atg5* mutant also exhibits defective microtubule organization during flagella formation, which may reflect a similar mechanism of cytoskeletal regulation by autophagy during spermiogenesis. Further investigation to identify targets of autophagy during spermiogenesis would be needed to understand the precise functions of autophagy during plant spermiogenesis.

## How Is Autophagy Involved in Plant Male Reproduction?

As described above, autophagy is involved in distinct male reproductive processes in land plants. However, the regulatory networks and precise targets of autophagy remain almost unknown. The first step to address this would be to determine whether autophagic degradation during male reproduction in each plant species is devoted to bulk degradation of the cytoplasm or selective degradation of certain targets. Recent studies have revealed that a wide range of targets, including organelles and proteins, are selectively degraded by autophagy in various organisms, including *A. thaliana* (Marshall and Vierstra, 2018; Johansen and Lamark, 2020). Selective autophagy appears to operate during spermiogenesis in plants because organelles unnecessary for spermatozoids seem to be removed through autophagic degradation (Minamino et al., 2017; Sanchez-Vera et al., 2017). Bryophyte spermatozoids only retain two mitochondria and a plastid in the cell body, potentially resulting from selective removal of unneeded organelles by autophagy. Furthermore, in germinating pollen of *N. tabacum*, mitochondrial markers are colocalized with an autophagosome marker, implying selective autophagic degradation of mitochondria (mitophagy) (Zhao et al., 2020). However, the existence of mitophagy is not firmly demonstrated in plants thus far (Broda et al., 2018) and detailed electron microscopic or super-resolution microscopic observation of phagophores and autophagosomes is needed to be conclusive. Genetic or pharmacological inhibition of autophagic body degradation in the vacuole would also be effective in investigating the targets of autophagic degradation during male reproduction. Another promising approach is to identify proteins that interact with ATG8, since ATG8 is involved in cargo recognition in selective autophagy as well as in the formation and transport of autophagosomes in various organisms (Nakamura and Yoshimori, 2017; Marshall and Vierstra, 2018; Mizushima, 2019; Stephani and Dagdas, 2019; Johansen and Lamark, 2020). Since many land plants possess multiple *ATG8* genes, each of which could play a specialized function (Kellner et al., 2017), it would be also informative to examine whether any of *ATG8* genes are highly and/or specifically expressed during male reproductive development.

Another enigma is how autophagy is regulated during male reproduction in plants. As described above, autophagic activity is highly activated in certain male reproductive processes. Autophagic activity can be regulated at several distinct levels, for example, at the transcriptional and post-transcriptional levels, as reported in *S. cerevisiae* and mammals (Fullgrabe et al., 2016; Corona Velazquez and Jackson, 2018). In *A. thaliana*, the expression of core *ATG* genes is spatiotemporally regulated, and the transcription factor TGA9 has been shown to positively regulate *ATG8* expression and autophagic activity (Slavikova et al., 2005; Rose et al., 2006; Wang et al., 2019). Post-transcriptional regulation has also been reported in *A. thaliana*, which is exemplified by that the TOR and SnRK1 complexes catalyze phosphorylation of and SINAT proteins mediate ubiquitylation of the ATG1 complex responding to the nutrient status (Chen et al., 2017; Huang et al., 2019; Van Leene et al., 2019; Qi et al., 2020). It would be useful to explore whether these regulations have a role in male reproduction in plants. Transcription factors responsible for the differentiation of male gametes have been identified in various organisms, including *M. polymorpha* (Hackenberg and Twell, 2019; Hisanaga et al., 2019). It would be worthwhile to study whether these transcription factors also regulate autophagic activities in order to understand the genetic regulation of autophagy during male reproduction.

## Author Contributions

TN and TU drafted the manuscript. NM edited the manuscript.

## Conflict of Interest

The authors declare that the research was conducted in the absence of any commercial or financial relationships that could be construed as a potential conflict of interest.
